# Wisconsin

**Published:** 1899-05

**Authors:** W. G. Clark

**Affiliations:** Secretary


Secretary.

### WISCONSIN SOCIETY OF VETERINARY GRADUATES.

THE annual meeting was held February 9, 1899, at Madison, in the rooms of the State Agricultural Society. The meeting was called to order at 2 P.M. by the President, Dr. B. L. Clark, Monticello. Present: Drs. B. L. Clarke, Monticello; W. G. Clark, Marinette; H. P. Clute, Marinette; L. A. Wright, Columbus; Charles Schmitt, Dodgeville; J. F. Roub, Monroe; G. Ed. Leech, Milwaukee; D. Roberts, Waukesha; R. S. Heer, Plattville; A. H. Hartwig, Watertown, and E. L. Morgenroth, Boltonville. Visitors: Dr. E. R. Flack, Manitowoc. The minutes of the last regular meeting were read and approved. The Secretary's report was read and accepted. The Treasurer's report was read and accepted.

Under the head of unfinished business the application of Dr. J. P. Laws for honorary membership was taken up. Moved by Dr. Clute and seconded by Dr. Hartwig, that the application be granted; carried.

The charges preferred against Drs. R. A. Higgins and John T. Unerth for a violation of the code of ethics, by holding office in a livestock insurance company and giving said company free veterinary services, were taken up and discussed by Drs. Hartwig, Leech, Clark, Schmitt, and Roberts. Moved and seconded that R. A. Higgins and John T. Unerth be expelled from the Association; carried.

The application for membership of Dr. E. R. Flack was presented and referred to the Board of Censors; they reporting favorably, the application was balloted on and Dr. E. R. Flack was declared elected to membership.

On motion a recess was taken for ten minutes.

When called to order the subject of veterinary legislation was taken up. The bill presented at the last session of the Legislature was read by the Secretary. Discussed by Drs. Hartwig, Leech, Clute, W. G. Clark, Schmitt, Flack, and Roberts.

Moved by Dr. Clark, and seconded by Dr. Leech, that the President appoint a committee of three to draw up an amendment to the present veterinary law, providing a penalty for violation of the same; carried. The President appointed as a committee Drs. Clute, Leech, and Roberts.

Dr. Charles Schmitt read a paper on "Parturient Apoplexy," in which he discussed the different pathological conditions in detail, and reported several cases and methods of treatment employed. Discussed by Drs. Heer, Roub, Clute, Hartwig, Roberts, and Leech.

On motion the Society adjourned to meet at 7.30 P.M.

*Evening Session.* The meeting was called to order at 7.30 by the President. The discussion of parturient apoplexy was resumed by Drs. Roub, Clute, Roberts, and Hartwig. On motion the essayist was excused and a vote of thanks tendered; carried.

Dr. R. S. Heer reported a case of "Fracture of the Os Pedis and Sloughing of the Hough," and after a time sloughing of the hoofs of the other three feet, probably due to laminitis from standing so long. On motion a vote of thanks was tendered Dr. Heer for his communication.

The plan of holding the semi-annual meeting was discussed. On motion, it was decided to hold the semi-annual meeting in Milwaukee.

On motion it was decided that the President and Secretary appoint one other member as a committee of arrangements for the semi-annual meeting.

The election of officers for the ensuing year resulted as follows: President, D. Roberts, Waukesha; Vice-President, J. F. Roub, Monroe; Secretary, W. G. Clark, Marinette; Treasurer, Charles Schmitt, Dodgeville; Censors: Drs. A. H. Hartwig, Watertown; H. P. Clute, Marinette, and R. S. Heer, Plattville.

It was moved and seconded that the members make a free-will offering of papers for the semi-annual meeting; carried.

H. P. Clute volunteered to read a paper on "Tuberculosis," A. H. Hartwig on "Parturient Apoplexy," also E. L. Morgenroth, C. Schmitt, and L. A. Wright, subjects to be chosen later.

On motion Dr. Wright was tendered a vote of thanks for his services as President.

The Secretary was instructed to mail a postal-card with each programme, that the committee on arrangements might know how large an attendance was to be expected.

On motion the Society adjourned to meet in Milwaukee in September, subject to the call of the President, Secretary, and Dr. G. Ed. Leech.

W. G. CLARK, M.D.C.,
Secretary.

